# Reduced repair of 8-hydroxyguanine in the human breast cancer cell line, HCC1937

**DOI:** 10.1186/1471-2407-6-297

**Published:** 2006-12-27

**Authors:** Simon G Nyaga, Althaf Lohani, Pawel Jaruga, Andrzej R Trzeciak, Miral Dizdaroglu, Michele K Evans

**Affiliations:** 1 Laboratory of Cellular and Molecular Biology, National Institute on Aging, National Institutes of Health, Baltimore, MD 21224, USA; 2 Department of Chemical and Biochemical Engineering, University of Maryland Baltimore County, Baltimore, MD 21250, USA; 3 Department of Clinical Biochemistry, Collegium Medicum, Nicolaus Copernicus University, Bydgoszcz, Poland; 4 Chemical Science and Technology Laboratory, National Institute of Standards and Technology, Gaithersburg, MD 20899, USA

## Abstract

**Background:**

Breast cancer is the second leading cause of cancer deaths in women in the United States. Although the causes of this disease are incompletely understood, oxidative DNA damage is presumed to play a critical role in breast carcinogenesis. A common oxidatively induced DNA lesion is 8-hydroxyguanine (8-OH-Gua), which has been implicated in carcinogenesis. The aim of this study was to investigate the ability of HCC1937 and MCF-7 breast cancer cell lines to repair 8-OH-Gua relative to a nonmalignant human mammary epithelial cell line, AG11134.

**Methods:**

We used oligonucleotide incision assay to analyze the ability of the two breast cancer cell lines to incise 8-OH-Gua relative to the control cell line. Liquid chromatography/mass spectrometry (LC/MS) was used to measure the levels of 8-OH-Gua as its nucleoside, 8-OH-dG in the cell lines after exposure to H_2_O_2 _followed by 30 min repair period. Protein expression levels were determined by Western blot analysis, while the *hOGG1 *mRNA levels were analyzed by RT-PCR. Complementation of hOGG1 activity in HCC1937 cells was assessed by addition of the purified protein in the incision assay, and *in vivo *by transfection of pFlagCMV-4-*hOGG1*. Clonogenic survival assay was used to determine sensitivity after H_2_O_2_-mediated oxidative stress.

**Results:**

We show that the HCC1937 breast cancer cells have diminished ability to incise 8-OH-Gua and they accumulate higher levels of 8-OH-dG in the nuclear genome after H_2_O_2 _treatment despite a 30 min repair period when compared to the nonmalignant mammary cells. The defective incision of 8-OH-Gua was consistent with expression of undetectable amounts of hOGG1 in HCC1937 cells. The reduced incision activity was significantly stimulated by addition of purified hOGG1. Furthermore, transfection of pFlagCMV-4-*hOGG1 *in HCC1937 cells resulted in enhanced incision of 8-OH-Gua. HCC1937 cells are more sensitive to high levels of H_2_O_2 _and have up-regulated SOD1 and SOD2.

**Conclusion:**

This study provides evidence for inefficient repair of 8-OH-Gua in HCC1937 breast cancer cell line and directly implicates hOGG1 in this defect.

## Background

Aerobic organisms are constantly exposed to endogenous and exogenous reactive oxygen species (ROS). Endogenous ROS include hydroxyl radicals that are produced predominantly as byproducts of normal cellular metabolism and through leakage of electrons during the mitochondrial electron transport chain. The interaction of ROS with the cellular genome often results in oxidatively induced DNA damage that includes; DNA base and sugar modifications, DNA-protein cross-links, abasic sites, single-strand breaks (SSB) and double-strand breaks (DSB) [1].

One of the most common oxidatively induced DNA base modifications is 8-hydroxyguanine (8-OH-Gua), which mispairs with adenine during DNA replication causing GC→TA transversion mutations [2,3]. To maintain proper genetic integrity and to minimize cancer risk, organisms ranging from *E. coli *to humans have evolved elaborate mechanisms for repairing 8-OH-Gua [4]. The MutT, MutM, and MutY pathways provide efficient repair of 8-OH-Gua in *E. coli *[5]. MutT and its human homologue hMTH is an 8-OH-dGTpase that cleanses the nucleotide pool of 8-OH-GuaTP thus preventing its incorporation into the DNA during DNA replication. MutY and its human homolog hMYH is a monofunctional DNA glycosylase that removes adenine opposite 8-OH-Gua. MutM (Fpg) is a DNA glycosylase/AP lyase that specifically removes 8-OH-Gua opposite cytosine. In humans, 8-OH-Gua is repaired predominantly by hOGG1, a DNA glycosylase/AP lyase that is specific for 8-OH-Gua and 2,6-diamino-4-hydroxy-5-formamidopyrimidine (FapyGua) opposite cytosine [6-9] via the base excision repair (BER) pathway [10,11].

A few studies have associated oxidatively induced DNA damage with cancer, [reviewed in [12]]. These include studies by Malins *et al*., showing increased hydroxyl radical-induced DNA damage in DNA of invasive ductal breast carcinoma tissues relative to normal breast tissue [13,14]. Olinski *et al*., showed that free radical-induced DNA damage is elevated in chromatin of various surgically removed malignant tissues from the colon, stomach, ovary, brain and lung relative to the surrounding non-malignant tissues [15]. In addition, peripheral blood lymphocytes of women at high risk of developing breast cancer have been shown to be deficient in the repair of X-irradiation-induced DNA damage [16]. Furthermore our laboratory recently showed that mitochondrial extracts but not nuclear extracts of breast cancer cell lines, MCF-7 and MDA-MB-468 are defective in the repair of 8-OH-Gua [17], consistent with increased levels of ROS in the mitochondria produced during the electron transport. It has also been shown that cell free extracts of HCC1937 cell line are defective in transcription-coupled repair (TCR) of 8-OH-Gua [18]. Here, we show that HCC1937 breast cancer cells are deficient in the repair of 8-OH-Gua when compared to nonmalignant mammary epithelial cells. We further show that HCC1937 breast cancer cells express undetectable levels of hOGG1. In addition, these cells accumulate elevated levels of 8-OH-Gua after H_2_O_2 _challenge despite a cellular repair period at 37°C. The repair of 8-OH-Gua was stimulated by addition of purified hOGG1 *in vitro *and *in vivo *by transient expression of hOGG1 in HCC1937 cells. This study directly implicates hOGG1 in the defective repair of 8-OH-Gua in HCC1937 breast cancer cells.

## Methods

### Cell lines and culture conditions

HCC1937 is a primary ductal breast carcinoma cell line that was purchased from American Type Culture Collection (ATCC Manassas, VA). The HCC1937 cells were cultured in RPMI 1640 media containing 10 mM HEPES, 1 mM sodium pyruvate, 2 mM L-glutamate, 4500 mg glucose/L, 1500 mg sodium bicarbonate supplemented with 10% FBS. MCF-7 is an epithelial breast adenocarcinoma cell line that was purchased from ATCC (Manassas, VA). MCF-7 cells were cultured in Minimum Essential Medium supplemented with 10% FBS, 1 mM sodium pyruvate, 2 mM L-glutamine, and 1500 mg sodium bicarbonate. The AG11134 is a nonmalignant human mammary epithelial cell line that was obtained from the Aging Cell Repository (Coriell Institute for Medical Research, Camden, NJ). The AG11134 cell line was cultured in serum-free mammary epithelial growth media (Cambrex, Walkersville, MD) supplemented with bovine pituitary extracts, human epidermal growth factor, insulin and hydrocortisone, gentamycin and amphotericin-B according to the supplier's instructions. All the cells had normal morphology and were harvested at viability greater than 95%.

### Preparation of nuclear extracts

Approximately 1.0 × 10^8 ^cells were harvested and pelleted by centrifugation at 585 × *g *at 4°C for 5 min using a tabletop centrifuge (Beckman Coulter, Allegra, 6R). The cell pellet was washed twice with PBS by centrifugation at 585 × *g *for 5 min at 4°C and the supernatant was saved for use in mitochondrial extract preparation. The cell pellet was lysed by homogenization (35 strokes) using a glass-glass dounce homogenizer (Thomas Scientific, Swedesboro, NJ) in buffer A (10 mM HEPES, pH 7.4; 0.21 M mannitol; 0.07 M sucrose; 0.15 M spermine; 0.75 M spermidine; 1 mM EDTA; 0.5 mM DTT) and protease inhibitors in the indicated final concentrations; 1 mM phenylmethylsulfonylfluoride (PMSF); 2 μM benzamide; 1 μg/mL chymostatinA; 1 μg/mL pepstatinA; 1 μg/mL aprotinin; 2 μg/mL leupeptin and 1 μM E64. The homogenate was spun at 500 × *g *for 7 min at 4°C (Beckman, Avanti J-25I) and the nuclear pellet was sonicated (Ultrasonic Processor GE130, Sonics & Materials, Inc., Newtown, CT) 3- times for 5 s each in half volume of buffer B (20 mM HEPES, pH 7.9; 25% glycerol; 0.2 mM EDTA; 0.5 mM DTT and 0.2 mM PMSF) on ice. The sonicated pellet was incubated on ice for 30 min before centrifugation for 30 min at 25,000 × *g *at 4°C (ultracentrifuge, Beckman XL-90). The supernatant was aliquoted and stored in -80°C. Total protein amounts were assessed by the Bradford assay (Bio-Rad, Hercules, CA).

### Preparation of whole cell and mitochondrial extracts

Whole cell extracts were prepared using approximately 1.0 × 10^8 ^cells as previously described [19]. Mitochondrial extracts were prepared from 1.0 × 10^8 ^cells following the procedure described earlier [20]. The nonmalignant cell line, AG10097 was used as a control for mitochondrial incision assay since it was difficult to grow enough of the primary AG11134 cells to prepare mitochondrial extracts for incision assay. The protein concentrations were assessed by the Bradford assay (Bio-Rad, Hercules, CA).

### Measurement of 8-OH-Gua in hydrogen peroxide-treated cells

Cells were grown in 150 × 25 mm tissue culture dishes in the respective media up to 85% confluency. The cells were washed twice with 1× PBS and treated with 300 μM H_2_O_2 _(Sigma-Aldrich, ST. Louis, MO) in 10 mL of 1× PBS. The control cells were treated with 10 mL of 1× PBS. The cells were incubated at 37°C, 5% CO_2 _and 95% relative humidity for 30 min. After this incubation, the H_2_O_2 _was removed and the cells were washed twice with 1× PBS. After this wash, 25 mL of media were added to the dishes and the cells incubated at 37°C for a further 30 min. After the 30 min incubation, the media was removed and the cells washed twice with 1× PBS. The cells were loosened by scrapping lightly and genomic DNAs were isolated by the salt extraction method [21]. The DNA samples were hydrolyzed to nucleosides using a mixture of three enzymes [22]. The hydrolysates were analyzed by liquid chromatography/mass spectrometry with isotope-dilution to quantitatively measure 8-OH-Gua as its nucleoside 8-hydroxy-2' -deoxyguanosine (8-OH-dG) using 8-OH-dG-^15^N_5 _as an internal standard as described [22].

### End-labeling of oligonucleotide

The 8-OH-Gua- or uracil-containing oligonucleotides had the following sequences:

5'-ATACGCATATACCGCT**8-OH-Gua**TCGGCCGATCTCCGAT-3', 5'-ATATACCGCGG**U**CGGCCGATCAAGCTTATT-3'. The positions of 8-OH-Gua and uracil (U) are shown in bold font. Approximately, 100 ng of the single-stranded oligonucleotides were 5' end-labeled with 30 μCi of [γ-32P] ATP (3,000 Ci/mmoL) using T4 polynucleotide kinase and annealed to 4-fold excess complementary oligonucleotides. The reactions were heated at 90°C for 10 min and then slow cooled to room temperature. The duplex oligonucleotides contained cytosine opposite 8-OH-Gua and guanine opposite uracil. Electrophoresis of the sample aliquots through 20% native polyacrylamide gel confirmed complete conversion of labeled single-stranded oligonucleotides to labeled duplex oligonucleotides.

### 8-OH-Gua incision assay

The 5' end-labeled duplex oligonucleotides were incubated with nuclear extracts at 37°C and the incision products analyzed qualitatively and quantitatively. The reactions were performed in buffer containing 20 mM HEPES, pH7.4, 5% glycerol, 0.1 mM NaCl, 5 mM EDTA, 5 mM DTT, 100 μg/mL BSA, and 25 μg/mL poly(dI)poly(dC). The hOGG1 reactions in the complementation of activity assay were performed following the procedure provided by the supplier of hOGG1 (Trevigen Inc., Gaithersburg, MD). All reactions were stopped by incubation with 0.2% SDS and 0.1 mg/mL proteinase K at 60°C for 15 min. The DNA was purified using phenol/chloroform/isoamylalcohol and the products separated by electrophoresis through 20% polyacrylamide gels containing 7 M urea at 15 W for 80 min. The gels were exposed in PhosphorImager cassettes and the gel images analyzed using PhosphorImager and ImageQuant version 5.1 (Amersham Biosciences, Piscataway, NJ). The product bands were quantified appropriately relative to the sum of the substrate and product bands and expressed as a percentage.

### Western blot analysis

The nuclear or whole cell extracts were mixed with sample loading buffer and then denatured by heating at 95°C for 10 min. The samples were loaded on a 6% or 12% Tris-glycine gels (Invitrogen, Carlsbad, CA) and separated by electrophoresis in 1X Tris-glycine SDS buffer. The proteins were subsequently transferred to PVDF membranes (Invitrogen, Carlsbad, CA). The membranes were blocked for 1 h in 5% dry milk in PBS-Tween (0.2%) and then probed with the following primary antibodies: OGG1 (Novus Biologicals, Littleton, CO), APE1 (Novus Biologicals), NEIL1 (Oncogene, San Diego, CA), p53 (Oncogene, San Diego, CA), SOD1 (Calbiochem, San Diego, CA), SOD2 (Abcam, Cambridge, MA) and catalase (Calbiochem). The membranes were washed and incubated with horseradish peroxidase-conjugated secondary antibodies and then developed using enhanced chemiluminescence plus Western detection kit (Amersham Biosciences, Piscataway, NJ). Loading errors were controlled using antibody against β-actin (Santa Cruz Biotechnology, Santa Cruz, CA).

### Stimulation of 8-OH-Gua specific activity in HCC1937 cells

Increasing amounts of hOGG1 were incubated at 37°C with approximately 20 ng of a duplex 5' end-labeled oligonucleotide containing a single 8-OH-Gua and 50 μg of nuclear extracts from HCC1937 in a 20 μl reaction. The reactions were heated to 90°C for 10 min and then 20 μl of oligonucleotide loading buffer containing 95% formamide, 5% glycerol and 0.02% bromophenol blue and 0.02% xylene cyanol was added. The samples were then separated by electrophoresis through 20% polyacrylamide gels containing 7 M urea. The band images were captured on phosphorImager cassettes and then analyzed by Imagequant software (PhorsphorImager).

### RNA isolation and RT- PCR

Total RNA was isolated from approximately 8.0 × 10^6 ^cells by the single-step method using TRIzol reagent (Invitrogen, Carlsbad, CA) according to the manufacturer's instructions. Briefly, 1 μg of total RNA was reverse-transcribed using 100 units MMLV RT (RETROscript kit, Ambion, Inc, Austin, TX) in a total volume of 20 μL. The hOGG1 cDNA was amplified for 22 cycles by PCR using the following primers: forward, 5'CCTACACCTCAGGAAAGCC 3' and reverse, 5' GGGAAGATAAAACCATCCTTAG 3'. β-actin was amplified for 30 cycles using commercial primers (Clontech Laboratories, Inc., Palo Alto, CA) and was used as a loading control. The amplification conditions used for both hOGG1 and β-actin cDNAs had been determined to be within the linear range of the product formation and also for detection on gels.

### Transient transfection of pFlag-CMV-4 with or without h*OGG1 *gene

HCC1937 cells (1.0 × 10^6^) were plated in several 100 mm × 20 mm tissue culture dishes in the relevant media without antibiotics and incubated at 37°C. After 24 h the cells were washed with 1× PBS and transfected with pFlag-CMV-4 plasmid carrying h*OGG1 *1a gene using DMRIE-C reagent (Invitrogen) according to the supplier's instructions. Nuclear and whole cell extracts were prepared thereafter. The expression of hOGG1 protein was examined by Western blot analysis using whole cell extracts and the incision activity was assayed using the nuclear extracts following the incision assay described above.

### Clonogenic survival

1.0 × 10^6 ^cells (AG11134, MCF-7 and HCC1937) were plated in triplicate in 100 mm × 20 mm dishes in their respective media and incubated at 37°C in the presence of 5 % CO_2_. After 24 h, the cells were washed twice with 1× PBS and treated with 0, 50, 150 and 300 μM of H_2_O_2 _in PBS for 30 min. The cells were then washed twice with 1× PBS and incubated at 37°C, 5% CO_2 _for 2 h in their respective media. The cells were then trypsinized and 2.5 × 10^4 ^cells were serially diluted. The diluted cells were re-plated in 100 mm × 20 mm dishes and incubated at 37°C for 20 days. The surviving colonies were quantified using MCID computer software.

### Statistical Analysis

Statistical analysis was performed using Statistica 6.1 (Statsoft Inc. Tulsa, OK). Data are presented as arithmetic means with error bars representing standard error of mean (SEM) calculated from at least three independent experiments. All statistical tests and corresponding *p*-values were two-sided. Differences between means were tested for significance using the student's t-test applying one- or two-way ANOVA. Where necessary post-hoc tests were performed by multiple comparisons between the experimental means relative to the control mean. A *p *value < 0.05 was regarded as statistically significant.

## Results

### 8-OH-Gua is poorly incised by HCC1937 nuclear extracts

**Figure 1:**
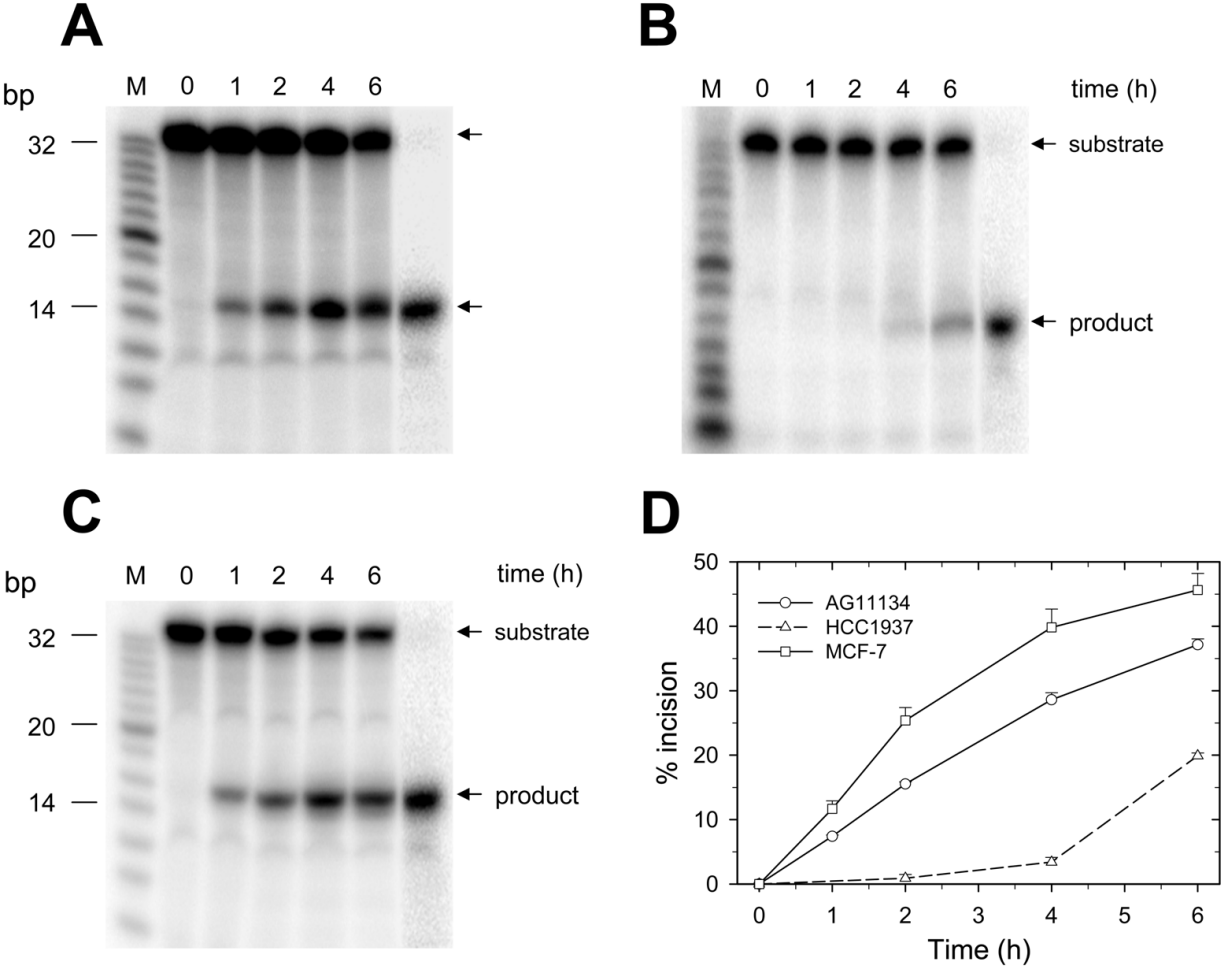
**Incision of 8-OH-Gua by AG11134 and HCC1937nuclear extracts**. 5' end-labeled duplex oligonucleotides containing single 8-OH-Gua were incubated at 37°C with 50 μg of nuclear extracts and aliquots removed at specified intervals as shown in the figure. The Fpg reactions were performed for 30 min. The reactions were stopped by the addition of 0.25% SDS and heating at 90°C for 10 min. The DNAs were purified by phenol/chloroform/isoamyl alcohol extraction method and then separated by electrophoresis through 20% denaturing gels. The Panels are: **A**, 8-OH-Gua incision by AG11134 nuclear extracts; **B**, 8-OH-Gua incision by HCC1937 nuclear extracts; **C**, 8-OH-Gua incision by MCF-7 nuclear extracts and **D**, quantified incision of 8-OH-Gua by AG11134, MCF-7 and HCC1937 nuclear extracts. The results are representative of three independent experiments and the error bars represent SEM.

**Figure 2:**
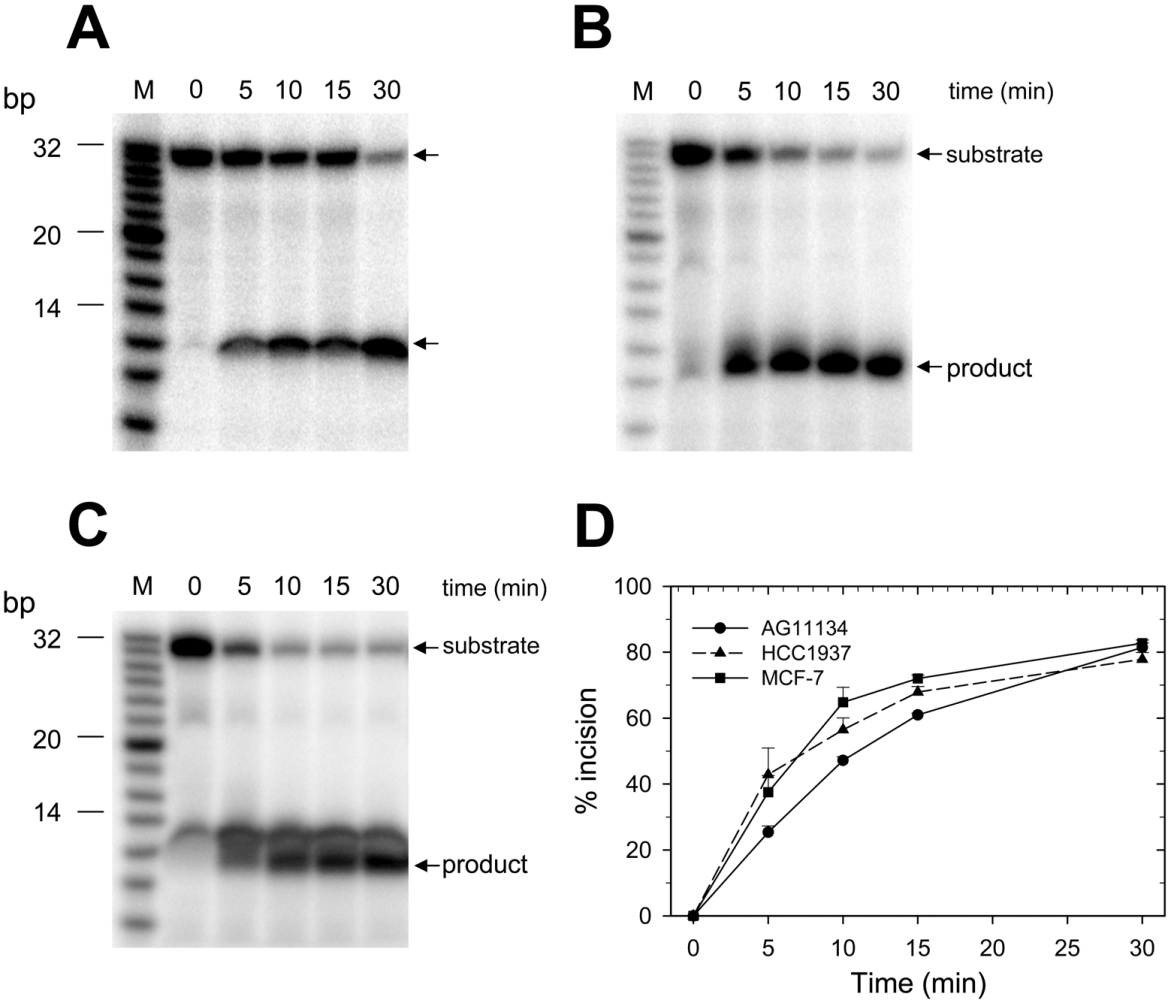
**Incision of uracil by AG11134 nuclear extracts**. The experiment was performed as in Fig. 1 except that uracil-containing oligonucleotide was used instead of 8-OH-Gua-containing oligonucleotide. Panel **A**, incision of uracil by AG11134 nuclear extracts, **B**, incision of uracil by HCC1937 nuclear extracts, **C**, incision of uracil by MCF-7 nuclear extracts and **D**, quantified incision of uracil by AG11134, HCC1937 and MCF-7 nuclear extracts. The results represent three independent experiments.

We tested the ability of nuclear extracts prepared from the breast cancer cell lines to incise an oligonucleotide containing 8-OH-Gua relative to the nonmalignant mammary epithelial cell line AG11134. HCC1937 nuclear extracts exhibited a statistically significant (*p *< 0.0002) diminished ability to incise an 8-OH-Gua-containing oligonucleotide compared to the nonmalignant mammary epithelial cell line, AG11134 (compare Fig. 1B with 1A, 1C and 1D, triangles with circles and squares). Nuclear extracts from MCF-7 incised 8-OH-Gua-containing oligonucleotide more effectively than the HCC1937 and the AG11134 cell lines. Fpg, a well-characterized DNA glycosylase/AP lyase incised the 8-OH-Gua-containing oligonucleotide yielding a product that had similar mobility with the products of incision by the extracts (Fig. 1A–C). These results suggest the presence of endonucleolytic activity of APE in the extracts. The incision of uracil by nuclear extracts from the two breast cancer cell lines, HCC1937 and MCF-7, was qualitatively and statistically similar to that of the nonmalignant mammary epithelial cell line, AG11134 (Fig. 2A–D). Thus, these results show that the breast cancer cell line HCC1937 is deficient in the incision of 8-OH-Gua. We noted that MCF-7 cells incised 8-OH-Gua relatively well probably due to the presence of detectable hOGG1 in the nuclear extracts of this cell line (Fig. 4A). A 13-mer band appeared in MCF-7 reactions (Fig. 2C). This band appears to be a nonspecific product since it did not change in the time course experiment.

### Measurement of 8-OH-Gua levels in genomic DNA

**Figure 3:**
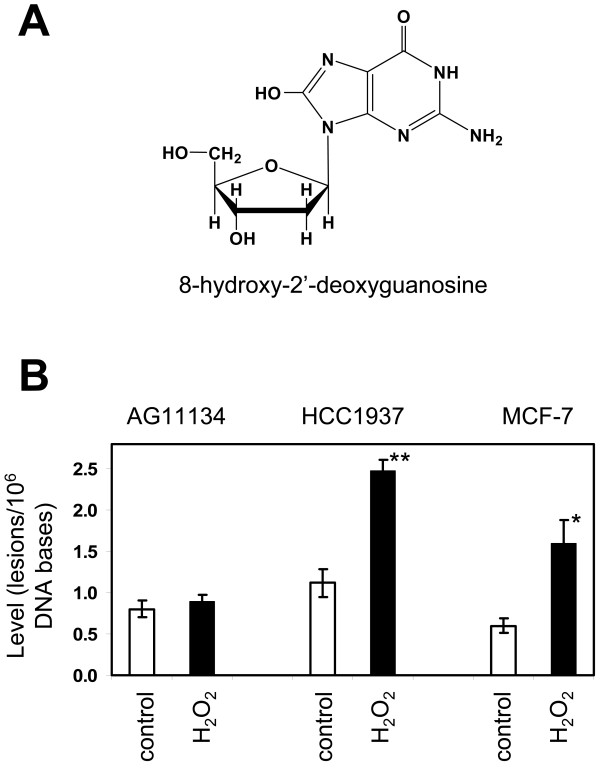
**Measurement of 8-OH-dG by LC/MS**. Levels of 8-OH-dG in genomic DNA of H_2_O_2_-treated and untreated samples were measured using LC/MS in genomic DNA. Panel **A **shows the chemical structure of 8-OH-dG and, panel **B **shows the measured levels of 8-OH-dG in untreated (open bars) and treated samples with 300 μM of H_2_O_2 _(solid bars). The results are representative of three independent experiments and the error bars represent standard deviation. Statistical analysis (t-test) was performed by comparing the means of the treated with untreated samples. * Represents a statistically significant value of *p *< 0.02, and ** represents a statistically significant value of *p *< 0.005.

In order to test the hypothesis that the breast cancer cell lines may be overwhelmed by oxidatively induced DNA damage after exposure to oxidative stress, we used LC/MS with isotope-dilution to measure the levels of 8-OH-Gua as its nucleoside 8-OH-dG in the genomic DNA of HCC1937, MCF-7 and AG11134 cells. The chemical structure of 8-OH-dG is shown in Fig. 3A. This analysis revealed that there was a statistically significant increase in the levels of 8-OH-dG in genomic DNA of HCC1937 and MCF-7 cells after exposure to 300 μM of H_2_O_2 _despite a 30 min repair period at 37°C (compare Fig. 3B, solid bars with open bars). There were no statistically significant differences between the levels of the 8-OH-dG in DNA of untreated AG11134 cells relative to H_2_O_2_-treated AG11134 cells (Fig. 3B), indicating complete repair of 8-OH-dG in this cell line. In contrast, H_2_O_2_-treated breast cancer cell lines accumulated significantly greater levels of 8-OH-dG, suggesting that the *in vivo *repair of 8-OH-Gua was deficient in the breast cancer cell lines, HCC1937 and MCF-7.

### Expression of DNA repair proteins and hOGG1 1a mRNA

**Figure 4:**
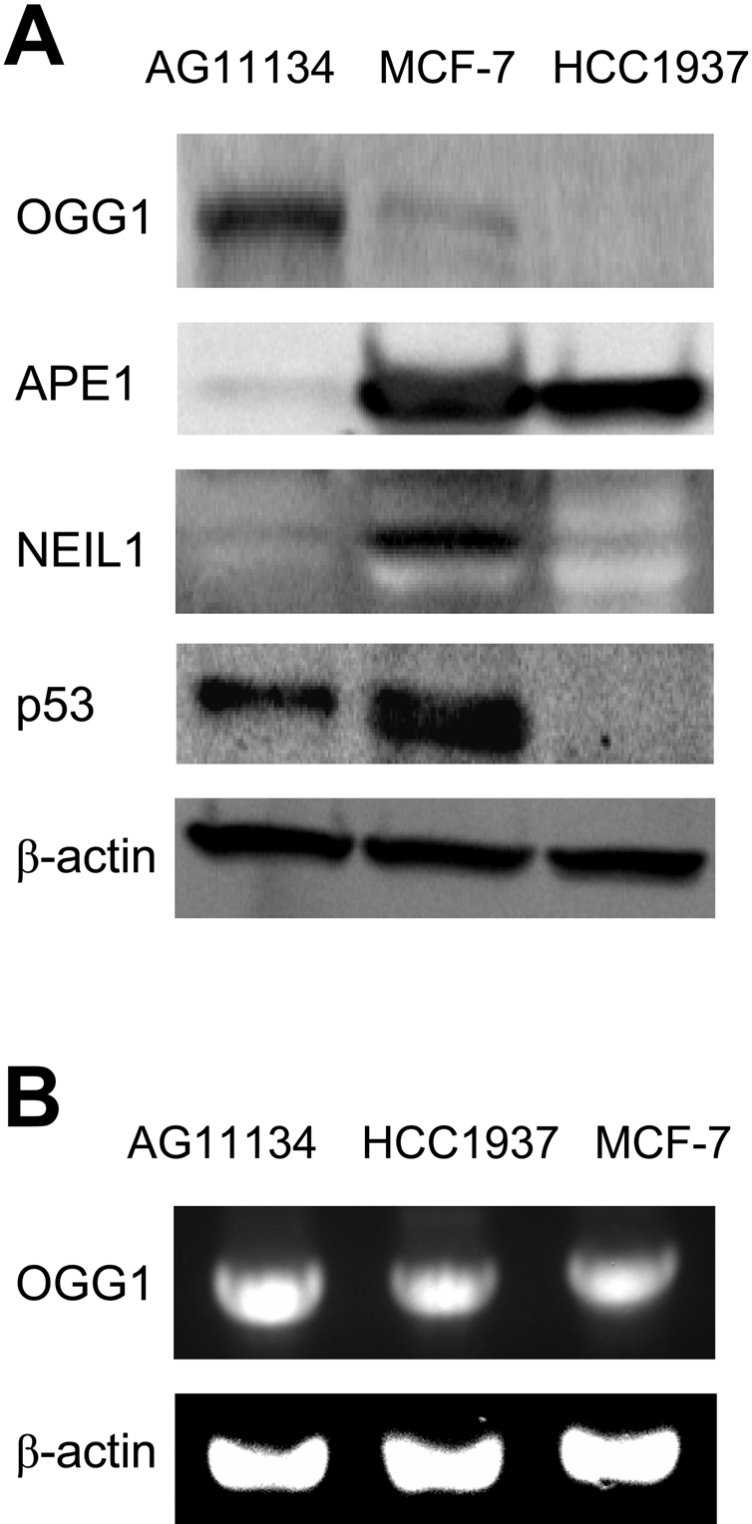
**Expression of specific DNA repair proteins**. Total proteins (50 μg) were separated by SDS-PAGE and the proteins transferred onto a PVDF membrane. The immunoblots were probed with antibodies against the specific proteins. Panel **A **represents basal levels of OGG1, NEIL1, p53 and APE1 in AG11134, MCF-7 and HCC1937 and, panel **B **shows RT-PCR showing the levels of OGG1 1a mRNA transcripts in AG11134, HCC1937 and MCF-7 cells. β-actin was used as a loading control.

To examine whether the reduced incision of the 8-OH-Gua-containing oligonucleotide is associated with the levels of hOGG1 and other proteins that influence DNA repair activity, we analyzed the expression of hOGG1, APE1, NEIL1 and p53 proteins in HCC1937 and MCF-7 relative to the control AG11134 cells. Western blot analysis showed that HCC1937 nuclear extracts had an undetectable level of hOGG1, while MCF-7 nuclear extracts did express active hOGG1 although at a slightly lower level than the control AG11134 nuclear extracts (Fig. 4A). APE1 was significantly upregulated in both HCC1937 and MCF-7 cells compared to AG11134 cells, whereas NEIL1 was over-expressed in MCF-7, but expressed at wild type level in HCC1937 cells. The p53 protein was undetectable in HCC1937 nuclear extracts but significantly over-expressed in MCF-7 nuclear extracts (Fig. 4A). These results suggest that the inefficient incision of 8-OH-Gua in HCC1937 cells could be due to undetectable levels of the hOGG1 in HCC1937.

In order to determine whether the reduced expression level of hOGG1 in the two breast cancer cell lines correlated with transcriptional regulation, we examined the expression of hOGG1 1a mRNA by RT-PCR. The results show that *OGG1 *1a gene was effectively transcribed in both HCC1937 and MCF-7 cells (Fig. 4B). These results were verified by real time PCR (data not shown).

### Stimulation of 8-OH-Gua repair in HCC1937 nuclear extracts by purified hOGG1

**Figure 5:**
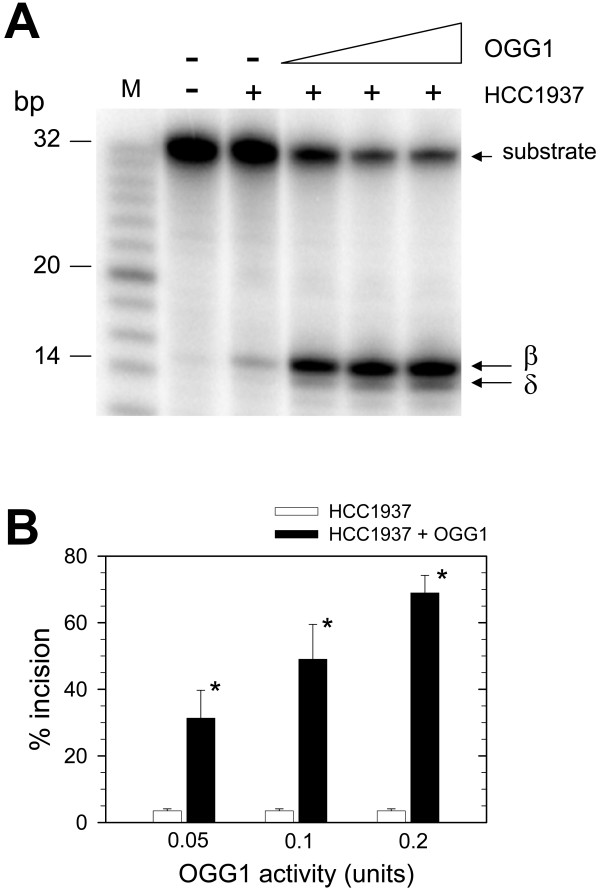
**Stimulation of 8-OH-Gua-specific incision activity of HCC1937 nuclear extracts by purified OGG1**. HCC1937 nuclear extracts (50 μg) were incubated with a 5' end-labeled oligonucleotide containing a single 8-OH-Gua in the presence of increasing amounts of hOGG1 for 1 h. Reactions were stopped and the products separated by electrophoresis through 20% polyacrylamide gels containing 7 M urea. The band images were analyzed by PhosphorImager and quantified using ImageQuant software. Panel **A**, a representative gel image showing the incision reaction products, panel **B**, quantified results of the incision reaction products. One-way ANOVA revealed a statistically significant difference of *p *< 0.002 (*) in the stimulation of activity by purified hOGG1. A unit of hOGG1 catalyzes the cleavage of 1 picomole of a duplex ^32^P-labeled oligonucleotide in 1 h at 37°C at 8-OH-Gua/C base pair.

Given that HCC1937 nuclear extracts exhibited diminished nicking activity of 8-OH-Gua and anti-hOGG1 antibodies did not detect hOGG1 in HCC1937, we speculated that addition of purified hOGG1 in HCC1937 extracts would restore the repair of 8-OH-Gua. Addition of purified hOGG1 to HCC1937 nuclear extracts resulted in significant stimulation of 8-OH-Gua repair in a concentration dependent manner resulting in the expected β-elimination and weak δ-elimination products (Fig. 5A and 5B). These results further prove that the reduced incision of 8-OH-Gua in HCC1937 cells is indeed related to the lack of hOGG1 in these cells.

### Transfection of *hOGG1 *in HCC1937 enhances 8-OH-Gua repair

**Figure 6:**
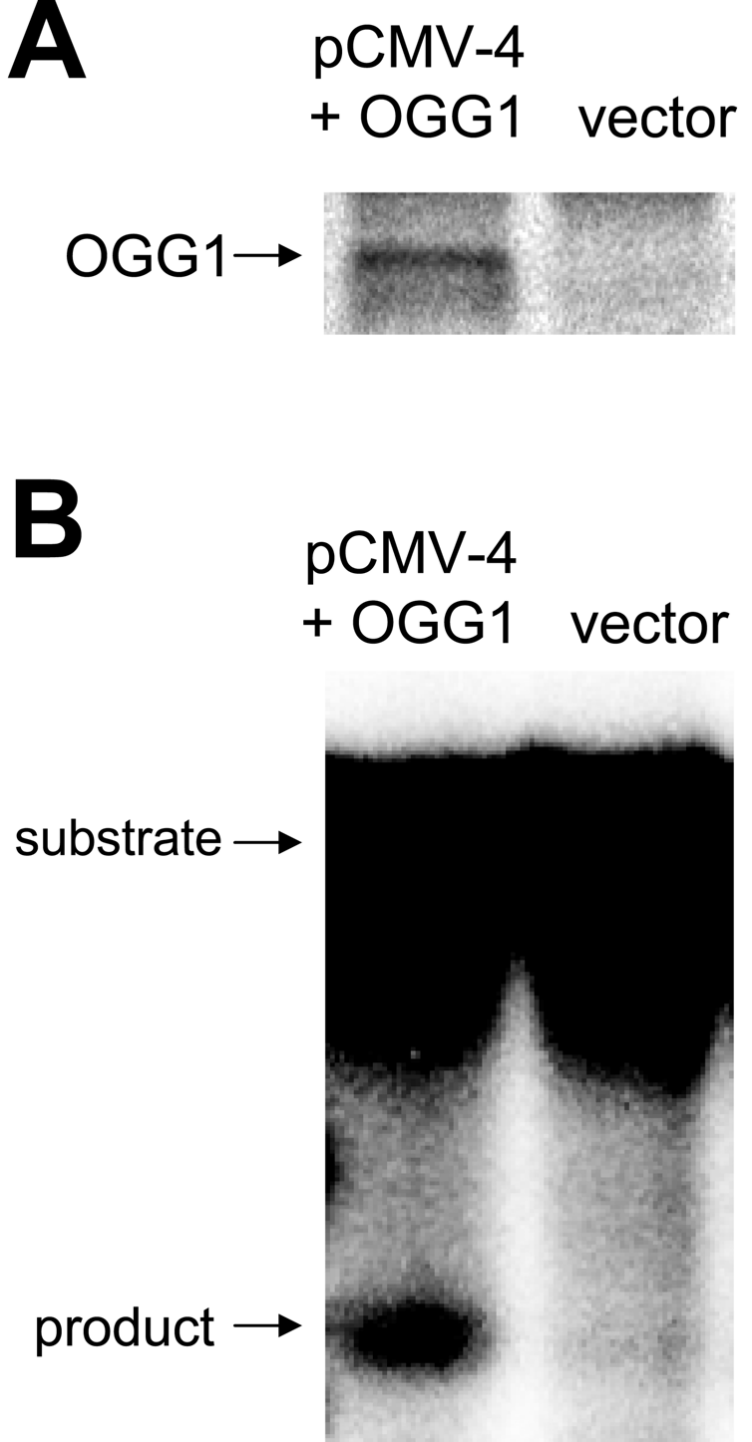
**Incision of 8-OH-Gua by HCC1937 nuclear extracts after transfection with pFlag-CMV-4-*OGG1***. HCC1937 cells (2.5 × 10^6^) were transfected with either pFlag-CMV-4 carrying *hOGG1 *gene or pFlag-CMV-4 without *hOGG1 *gene and nuclear extracts and whole cell extracts were prepared 48 h after transfection. Western blot analysis was performed using whole cell extracts and incision reactions performed using nuclear extracts and a duplex 5' end-labeled oligonucleotide containing a single 8-OH-Gua. Panel **A**, shows the expression of pFlag-CMV-4-OGG1 in HCC1937 cells and, panel **B**, shows the incision of 8-OH-Gua by nuclear extracts containing the transfected pFlag-CMV-4-OGG1 or just the vector alone.

In order to further ascertain that lack of hOGG1 contributed to the reduced *in vivo *repair of 8-OH-Gua in HCC1937 cells, we transiently transfected the *hOGG1 *gene into HCC1937 cells. Western blot analysis revealed that HCC1937 cells transfected with a plasmid carrying the *hOGG1 *gene expressed detectable levels of hOGG1, while HCC1937 cells transfected with the plasmid alone (without *hOGG1 *gene) expressed undetectable amounts of hOGG1 (Fig. 6A). Incision assay showed that nuclear extracts prepared from HCC1937 cells expressing hOGG1 exhibited higher 8-OH-Gua-specific incision activity, while nuclear extracts from cells transfected with the vector alone had limited or no excision repair activity (Fig. 6B).

### Altered expression of antioxidant enzymes in HCC1937 cells

**Figure 7:**
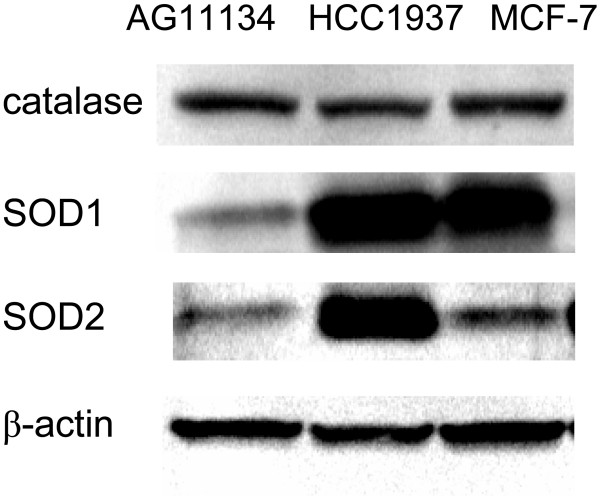
**Expression of antioxidants in HCC1937 and MCF-7 relative to AG11134**. Western Blot analysis of SOD1, SOD2 and catalase showing their levels in the two breast cancer cell lines compared to the normal mammary epithelial cell line, AG11134. β-actin was used as a loading control. The gels are representatives of three independent analyses in each case.

Cells express antioxidant enzymes as a means of extra protection against oxidatively induced DNA damage. We therefore analyzed the level of antioxidant enzymes, SOD1, SOD2 and catalase in HCC1937 and MCF-7 nuclear extracts relative to the control extracts. Interestingly, the level of SOD1 was considerably increased in both breast cancer cell lines, HCC1937 and MCF-7 (Fig. 7). The mitochondrial antioxidant enzyme, SOD2, was over-expressed in HCC1937 cells, whereas catalase was expressed at wild type level in both breast cancer cell lines (Fig. 7).

### Reduced clonogenic survival of HCC1937 cells after oxidative stress

**Figure 8:**
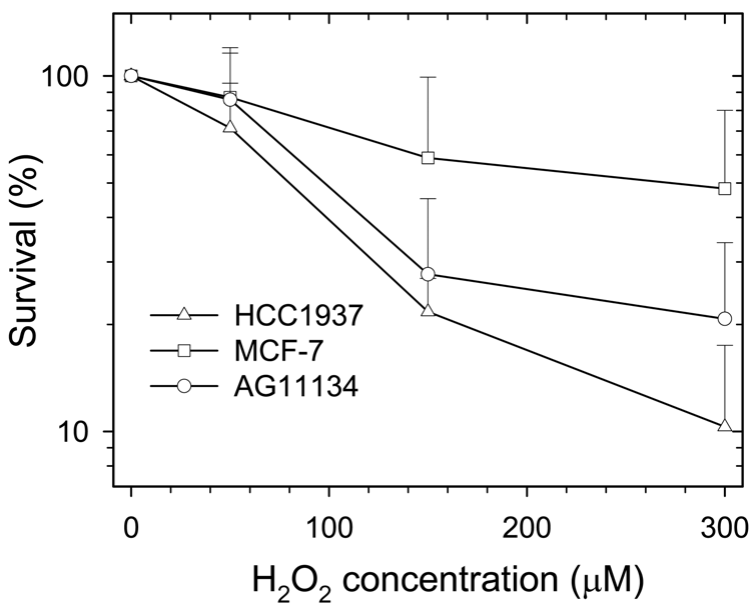
**Clonogenic survival to oxidative stress**. 1.0 × 10^6 ^cells were grown for 24 h in 37°C and 5% CO_2 _incubators. Following this incubation, the cells were washed with 1× PBS and treated with H_2_O_2 _in PBS for 30 min. The cells were washed twice with 1× PBS and incubated at 37°C, 5% CO_2 _in their respective media for 2 h. Serial dilutions of the cells were made in media and the dilutions were plated. The cells were then incubated at 37°C, 5% CO_2 _for 20 days. The cells were washed twice with 1× PBS and then stained with 0.02% crystal violet. The surviving colonies were scored using MCID computer software. The symbols are, diamonds for AG11134, squares for MCF-7 and triangles for HCC1937. The experiments were performed in triplicates and the error bars represent standard error of mean.

In order to determine whether the increased level of oxidatively induced DNA damage and upregulation of SOD1 and SOD2 affected cellular survival, we analyzed the clonogenic survival after oxidative stress induction by H_2_O_2_. Our results show that HCC1937 cell line was more sensitive to high concentrations of H_2_O_2 _compared to both AG11134 and MCF-7 cell lines (Fig. 8). Conversely, MCF-7 cells were more resistant to H_2_O_2 _than either the HCC1937 or the AG11134 cells. These results suggest that HCC1937 cells and the MCF-7 cells use different mechanisms to survive H_2_O_2_- mediated stress. These results further suggest that oxidatively induced DNA damage repair and misregulation of antioxidants SOD1 and SOD2 may play a role in influencing the clonogenic survival of cells to H_2_O_2_-induced oxidative stress. Furthermore, these results suggest that the limited survival of HCC1937 cells may be partly due to the up-regulation of SOD1 and SOD2.

## Discussion

We report here that HCC1937 breast cancer cell line is deficient in the *in vitro *and *in vivo *repair of 8-OH-Gua, compared to the nonmalignant mammary epithelial cell line, AG11134. An incision assay showed that HCC1937 nuclear extracts had significantly reduced ability to incise a duplex 8-OH-Gua-containing oligonucleotide compared to the control AG11134 nuclear extracts (Fig. 1A–D). This deficiency correlated with lack of expression of the hOGG1 in HCC1937 cells (Fig. 4A). Defective repair of 8-OH-Gua is not a unique characteristic of HCC1937 cells since defective repair of this lesion has been reported in other cell types. To this end, our laboratory has previously reported a deficiency in the repair of 8-OH-Gua in mitochondria of the breast cancer cell lines, MCF-7 and MDA-MB-468 [17]. Interestingly, these cell lines exhibited normal nuclear repair of 8-OH-Gua and expressed normal levels of both nuclear and mitochondrial forms of the hOGG1. Furthermore, we recently showed that two prostate cancer cell lines, PC-3 and DU-145 are deficient in the nuclear and mitochondrial repair of 8-OH-Gua [19]. These cell lines expressed normal levels of the nuclear OGG1 but significantly low levels of the mitochondrial OGG1. Colorectal cancer cell lines have also been shown to be defective in the repair of 8-OH-Gua [23]. It has also been shown that Cockayne syndrome cells are deficient in 8-OH-Gua [24-26]. In addition, deficient 8-OH-Gua repair has been reported in lung cancer cell lines with reduced hOGG1 mRNA and protein expression [27]. Reduced 8-OH-Gua repair has recently been reported in isogenic cell lines (HCT116 p53^+^/^+ ^and HCT116 p53^-^/^-^) exhibiting reduced hOGG1 mRNA and protein expression after p53 down-regulation and oxidative stress challenge [28]. Furthermore, HCC1937 cell-free extracts are defective in transcription-coupled repair of 8-OH-Gua [18]. The studies discussed above along with the present study indicate that 8-OH-Gua repair is critical in different cell types and implicate hOGG1 levels/activity in certain forms of cancer. The stability of *hOGG1 *1a mRNA in HCC1937 is not known and may as well be important in the defective repair. On the other hand, HCC1937 cells were proficient in the mitochondrial repair of 8-OH-Gua (data not shown). Interestingly, the mitochondrial repair of 8-OH-Gua was more effective in HCC1937 cells than the normal cell line at higher time points (data not shown), suggesting that OGG1 2a is more active than the OGG1 1a in HCC1937 cells.

We noted that MCF-7 nuclear extracts incised 8-OH-Gua at a higher rate than the HCC1937 and AG11134 nuclear extracts, probably due to expression of hOGG1 and better stimulation of OGG1 activity resulting from a higher expression level of APE1 and NEIL1 compared to AG11134 and HCC1937 cells (Fig. 4A). This may be the case since APE1 and NEIL1 have been shown to functionally interact and to stimulate the activity of hOGG1 [29-32]. The deficient repair of 8-OH-Gua in HCC1937 cells may not be associated with low APE1 or NEIL1 expression since APE1 expression is up-regulated in HCC1937 cells and NEIL1 is expressed at wild type level in HCC1937 cells (Fig. 4A). This is in agreement with the evidence that hNEIL1 as well as mouse NEIL1 do not remove 8-OH-Gua from DNA [33]. The defective repair is most likely lesion-specific rather than a generalized BER defect since HCC1937 nuclear extracts repaired uracil at levels comparable to AG11134 extracts (Fig. 2 panels A-D). The efficient uracil repair may be attributed to the ubiquitous nature of uracil DNA glycosylase (UDG), the main enzyme involved in the repair of uracil, and also due to the redundancy of uracil excision repair [34]. The incision of uracil provided another control for extract activity.

The breast cancer cell lines, HCC1937 and MCF-7 accumulated statistically significant levels of 8-OH-Gua (measured by LC/MS as its nucleoside 8-OH-dG) after induction of oxidative stress by treatment with H_2_O_2 _despite allowing the cells time to repair the DNA damage (Fig. 3B). This accumulation of 8-OH-Gua may be a consequence of inefficient *in vivo *repair of 8-OH-Gua in the case of HCC1937 cells. This implies that HCC1937 cells may be sustaining an overwhelming level of ROS-induced DNA damage endogenously, a situation that could be exacerbated by exogenous oxidative stress challenge by treatment with H_2_O_2 _and the lack of hOGG1. Consistent with our findings there is evidence that inactivation of *Fpg*, *MutT *and *Ogg1 *resulted in increased levels of 8-OH-Gua that correlated with expression of mutator phenotypes in *E. coli *and yeast [3,35]. *Ogg1 *knockout mice exhibit increased levels of 8-OH-Gua in their livers [36-38]; however, these animals have only a modest increase in mutation frequency, probably due to DNA repair pathway redundancies. The LC/MS data indicate that the MCF-7 cell line is defective in the repair of 8-OHGua (Fig. 3B); however, the incision assay shows that this cell line repairs 8-OH-Gua effectively (Fig. 1C and 1D). These varied observations are not surprising since the LC/MS data are reflective of the more *in vivo *situation, whereas the incision assay is semi-*in vivo *that utilizes an oligonucleotide with only one lesion at a defined position. On the contrary, the LC/MS data are reflective of multiple 8-OH-Gua lesions that are introduced in the genomic DNA by H_2_O_2 _treatment of the cells. Our 8-OH-Gua measurements in the breast cancer cells are consistent with those reported for frozen breast tissues of women with breast cancer compared with normal breast tissue of women undergoing reduction mamoplasty [39].

The correlation between reduced repair of 8-OH-Gua and lack of OGG1 in HCC1937 cells would imply that adding the purified enzyme or enhancing its expression by transfection of the *hOGG1 *gene, would result in stimulation of the repair activity. The ability of purified hOGG1 to stimulate the 8-OH-Gua-incision activity of HCC1937 extracts (Fig. 5A and 6B) indicates that hOGG1 is required for efficient repair of 8-OH-Gua in these cells. The *in vivo *transient expression of a plasmid carrying *hOGG1 *gene in HCC1937 cells (Fig. 6A) resulted in 8-OH-Gua incision activity greater than the activity in extracts prepared from HCC1937 cells transfected with the vector alone (Fig. 6B). This *in vitro *and *in vivo *stimulation of 8-OH-Gua-repair by either the purified hOGG1 or the expressed hOGG1 after transfection of the *hOGG1 *gene directly implicates hOGG1 in the defective repair of 8-OH-Gua and suggests that the cellular levels of hOGG1 may be related to the functional activity of this enzyme in HCC1937 cells. These results are consistent with a number of studies that have clearly shown that defective mitochondrial repair in HeLa cells and pulmonary artery endothelial cells could be enhanced significantly by transfection with a plasmid carrying mitochondrial *hOGG1 *gene [40,41].

Altered enzymatic activity of the hOGG1 can result from mutations in the corresponding gene. This is supported by a report showing that a guanine to adenine mutation in the *hOGG1 *gene changes Arg46 to Gln46, and this amino acid change results in production of a hOGG1 with reduced activity [42]. Furthermore, the polymorphic hOGG1 (S326C) has reduced incision activity of 8-OH-Gua and is associated with cancer in certain human populations [9,43]. Our data on the sequence analysis of all the exons and the promoter region of the *hOGG1 *gene in HCC1937 cells did not reveal any mutations (data not shown), suggesting that the repair defect was not due to a genetic change.

The survival of cells to genotoxic stress is influenced by a number of factors including antioxidant enzymes. The upregulation of antioxidants SOD1 and SOD2 that we observed in HCC1937 cells (Fig. 7) may be an indication of increased oxidatively induced stress in these cells. The increased stress may in turn trigger a mechanistic response involving up-regulation of SOD1 and SOD2 that in turn reduces the toxicity of superoxide anion thereby confering some cellular survival. The high level of SOD2 in the mitochondrial extracts of HCC1937 cells is consistent with high metabolic activity in this organelle and may occur in response to accumulation of high levels of ROS and increased DNA damage in the mitochondrial genome [44].

The absence of hOGG1 in HCC1937 cells implies that these cells may be highly susceptible to oxidatively induced DNA damage. Indeed, we observed that HCC1937 cells were more sensitive to H_2_O_2 _treatment compared to MCF-7 and AG11134 cells at high H_2_O_2 _levels (Fig. 8). This sensitivity correlates with the lack of hOGG1 (Fig. 4A) and is probably exacerbated by increased oxidatively induced DNA damage (Fig. 3B) in this cell line. The reduced clonogenic survival of HCC1937 cells after H_2_O_2 _challenge relative to MCF-7 and AG11134 cells (Fig. 8) is consistent with the previously reported high degree of sensitivity of these cells to H_2_O_2 _treatment in the presence of DNA repair inhibitors [45]. Furthermore, HCC1937 cells were shown to be hypersensitive to ionizing radiation, a phenomenon that was reversed by expression of non-growth suppressing forms of BRCA1 [18]. In addition, single cell gel electrophoresis (comet) assay revealed that HCC1937 cells accumulate high levels of SSB and DSB relative to the control cells after exposure to 10 Gy of ionizing radiation followed by a repair period (data not shown). MCF-7 cells appear to be more resistant to oxidative stress than HCC1937 cells probably due to their effectiveness in incising 8-OH-Gua (Fig. 1C and 1D).

A monoclonal antibody against p53 did not detect any appreciable p53 protein in HCC1937 cells while this protein was over-expressed in MCF-7 cells relative to the control AG11134 (Fig. 4A). The lack of detectable p53 in HCC1937 cells may suggest a role for this protein in the repair of 8-OH-Gua in HCC1937 cells. Recently, Achanta and Huang used p53 isogenic cell lines HCT116 p53^+^/^+ ^and HCT116 p53^-^/^- ^to show that the former cell line excised 8-OH-Gua more rapidly than the latter [46]. Chatterjee *et al*., recently confirmed these findings using the same isogenic cell lines by showing that HCT116 p53^-^/^- ^exhibited significant reduction in OGG1 activity [28]. This same group further showed that down-regulation of p53 resulted in decreased expression of OGG1 mRNA/protein after oxidative DNA damage induction and that these events correlated with reduced incision of 8-OH-Gua. Taken together these studies suggest that the defective repair of 8-OH-Gua in HCC1937 cells may be associated with complex protein interactions involving OGG1 and other proteins.

The likelihood of BRCA1 playing a role in the repair of 8-OH-Gua in HCC1937 cells cannot be ruled out especially since HCC1937 cell line harbors a C-terminally truncated BRCA1 due to a 5382C insertion mutation on the *BRCA1 *gene [47]. In contrast, the MCF-7 cell line carries a wild type BRCA1 and our studies showed that it incised the 8-OH-Gua-containing oligonucleotide quite effectively (Fig. 1 panel C, and panel D, open squares). A possible role for BRCA1 in oxidative DNA damage repair could be achieved through direct interactions with proteins that repair DNA damage. Alternatively, such a role can occur indirectly through interactions with proteins that regulate the stability or expression of the DNA repair proteins. It is noteworthy that the deleted carboxyl terminal domain of BRCA1 in HCC1937 contains the BRCT domain that is thought to mediate BRCA1 interactions with other DNA interactive proteins [48] and is also found in many other proteins involved in DNA repair and DNA damage cell cycle check point control [49-51]. Furthermore, mutant murine *Brca1 *and mutant human BRCA1 cells are hypersensitive to oxidative DNA damaging agents such as ionizing radiation and hydrogen peroxide [18,52].

Thus, we have shown that HCC1937 breast cancer cell line is deficient in the incision of 8-OH-Gua and accumulates significantly high levels of this lesion after H_2_O_2 _treatment followed by a repair period. This cell line does not express any detectable level of hOGG1, linking this enzyme to the inefficient 8-OH-Gua-repair. The DNA repair deficiency was stimulated *in vitro *by addition of purified hOGG1. Furthermore, the transfection of a plasmid carrying the *hOGG1 *1a gene and *in vivo *expression of hOGG1 in HCC1937 cells resulted in increased incision of 8-OH-Gua. HCC1937 cells were more sensitive to high H_2_O_2 _concentrations compared to both AG11134 and MCF-7 cells. This work implicates hOGG1 in the defective repair of 8-OH-Gua in HCC1937 cells and suggests that oxidatively induced DNA damage is a critical factor in breast cancer.

## Conclusion

This research has shown that HCC1937 breast cancer cells are deficient in the repair of 8-OH-Gua. The increased expression of hOGG1 and enhanced incision of 8-OH-Gua in HCC1937 cells after transfection with a plasmid carrying the *hOGG1 *directly implicates hOGG1 in the defective repair of 8-OH-Gua. This defect is underscored by the accumulation of statistically significant levels of 8-OH-dG in the breast cancer cells after H_2_O_2_-mediated oxidative stress relative to the nonmalignant mammary cell line despite a DNA repair period. Taken together, these findings implicate defective 8-OH-Gua repair in certain breast cancer cell lines and suggest that oxidative DNA damage could be a risk factor in the development/progression of a proportion of breast cancers.

## Abbreviations

BER, base excision repair; ROS, reactive oxygen species; LC/MS, liquid chromatography/mass spectrometry; GC/MS, gas chromatography/mass spectrometry; 8-OH-Gua, 8-hydroxyguanine; 8-OH-dG, 8-hydroxy-2'-deoxyguanosine; TCR, transcription-coupled repair; SSB, single-strand breaks; DSB, double-strand breaks.

## Competing interests

The author(s) declare that they have no competing interests.

## Authors' contributions

SGN co-designed the study with MKE, wrote the manuscript, prepared extracts, performed incision assays, treated and isolated genomic DNA for 8-OH-dGua measurements, performed transfection and DNA repair complementation studies, RT-PCR and clonogenic survival assays. AL performed cell culture work, Western blot analysis and participated in incision studies, RT-PCR and clonogenic survival studies. PJ and MD measured the 8-OH-dG levels by LC/MS and contributed in part to the preparation of the manuscript. ART performed the statistical analysis and assisted in figure preparation. MKE co-designed the study with SGN, coordinated, supervised the study and edited the manuscript. All the authors read and approved the final manuscript.

## Pre-publication history

The pre-publication history for this paper can be accessed here:
